# Phase II study of a triplet regimen in advanced colorectal cancer using methotrexate, oxaliplatin and 5-fluorouracil

**DOI:** 10.1038/sj.bjc.6602176

**Published:** 2004-09-21

**Authors:** A Guglielmi, S Barni, A Zaniboni, N Pella, O Belvedere, G D Beretta, F Grossi, L Frontini, F Puglisi, R Labianca, A Sobrero

**Affiliations:** 1 Medical Oncology, Ospedale San Martino, Genova, Italy; 2 Medical Oncology, Ospedale di Treviglio, Treviglio, Italy; 3 Medical Oncology, Spedali Civili di Brescia, Brescia, Italy; 4 Medical Oncology, Ospedale S Maria, Misericordia, Udine, Italy; 5 Medical Oncology, Università di Udine, Udine, Italy; 6 Medical Oncology, Ospedali Riuniti di Bergamo, Bergamo, Italy; 7 Division of Medical Oncology, Ospedale S Pio X, Milano, Italy

**Keywords:** chemotherapy, colorectal cancer, 5-fluorouracil, methotrexate, oxaliplatin

## Abstract

Building upon the concept of schedule-specific biochemical modulation of 5-fluorouracil (FU), which alternates bolus and continuous infusion (CI) FU, we have incorporated oxaliplatin (l-OHP) following the bolus part of the regimen to explore the activity of this new combination. Patients with advanced, untreated, measurable colorectal cancer received sequential methotrexate (MTX) (days 1 and 15) → l-OHP+FU (days 2 and 16) (200, 85 and 600 mg m^−2^, respectively) followed by 3 weeks of CI FU (200 mg m^−2^ day^−1^) given from day 29 to 50, modulated by weekly leucovorin (LV) (20 mg m^−2^). After 1 week of rest, the second cycle was started. The treatment was continued until progression or patient's refusal. According to the intention-to-treat analysis on all 46 patients accrued, the response rate was 42% (95% CL=28–55%), with three complete responses and 16 partial responses. The median overall survival was 15.9 months and the median progression-free survival 6.9 months. Toxicity was very mild, with the bolus part of the regimen more toxic than the infusional part (24 *vs* 7% of grade III–IV, respectively). This new combination of MTX → l-OHP−FU followed by FU CI is well tolerated, feasible and produces activity results similar to other more simple, but more toxic, regimens. Pros and cons of the different fluoropyrimidines–l-OHP combinations are discussed.

Chemotherapy, both first and second and possibly third line, prolongs survival as compared to best supportive care in the treatment of advanced colorectal cancer (Cunningham and Glimelius, 1999; Jonker *et al*, 2000; Baldwin, 2002). Doublets (either 5-fluorouracil (FU)+irinotecan or FU+oxaliplatin (l-OHP)) prolong survival in first and second line as compared to modulated bolus FU (de Gramont *et al*, 2000; Douillard *et al*, 2000; Levi *et al*, 2001; Baldwin, 2002; Saltz *et al*, 2000) and it has recently emerged that giving ‘doublets’ means using infusional FU with either irinotecan or l-OHP; otherwise, these combinations are too toxic (Grothey *et al*, 2002; Ravaioli *et al*, 2002; Saltz *et al*, 2000; Goldberg *et al*, 2003). Doublets are more efficacious but more toxic than FU alone, so it is uncertain whether it is better to start with a doublet or to use the drugs sequentially; in addition, doublets are more efficacious than FU alone only in patients under age 70, with a good performance status (PS) and relatively normal serum chemistries (Knight *et al*, 2000). The scenario of advanced colorectal cancer treatment is completed by the oral fluoropyrimidines, which may be a valid alternative to FU because they are substantially equivalent to bolus FU but are preferred by patients (Liu *et al*, 1997; Borner *et al*, 2000; Cassidy *et al*, 2002), and by targeted therapies, promising, but still highly experimental (Saltz *et al*, 2001; Cunningham *et al*, 2002; Giantonio *et al*, 2002; King *et al*, 2002).

We have published four clinical trials based upon our hypothesis that FU is indeed two different drugs depending upon the schedule of administration (Sobrero *et al*, 1993, 1995, 2000, 2001; Aschele *et al*, 1998): maximal enhancement of bolus FU is more likely obtained with drugs that enhance the RNA effect of the fluoropyrimidine such as methotrexate (MTX), while leucovorin (LV), which selectively enhances the TS^*^ inhibitory activity of FU, may result in greater potentiation when the fluoropyrimidine is administered as continuous infusion (CI) (Aschele *et al*, 1998). We are now in the process of integrating the new agents irinotecan and l-OHP in our basic regimen. In particular, we added l-OHP to MTX → FU bolus, leaving the infusional part unchanged. Preclinical data on four human colorectal cancer cell lines suggest that l-OHP synergises more with short-term exposure to FU than with prolonged FU exposures (Fischel *et al*, 1998). In addition, our previous experience with this regimen without l-OHP (Sobrero *et al*, 1995) indicated that the toxicity of the MTX → FU part was much lower than that of the CI FU part. For this reason, we decided to add l-OHP to the first part of the regimen. Preliminary to this study, we explored the feasibility of administering full-dose l-OHP 130 mg m^−2^, but observed prohibitive toxicity; therefore, we chose 85 mg m^−2^ of the diaminocyclohexane (DACH) platinum compound.

## PATIENTS AND METHODS

### Eligibility criteria

Patients enrolled in the study had histologically confirmed, unresectable metastatic colorectal cancer and bidimensionally measurable disease. No prior chemotherapy for metastatic disease was allowed, while adjuvant chemotherapy was permitted if completed at least 6 months before study entry. Radiation therapy was allowed as long as it did not encompass the indicator lesions. All patients had an ECOG PS ⩽2. Adequate bone marrow, liver and renal functions were required.

Additional eligibility criteria included geographic accessibility, the absence of clinically relevant ascites and the absence of other medical conditions clearly contraindicating the delivery of any chemotherapy.

Informed consent was required. Before treatment, patients were informed as to (a) the presence of metastatic colorectal cancer, (b) the poor prognosis of their disease and (c) the experimental nature of this treatment protocol. Upon study entry, all patients were given a schedule of drug treatment along with written information about the anticipated toxicities.

### Treatment plan

The regimen consisted of the alternating regimen of bolus and infusional FU that we previously tested in a phase II trial and that was derived from two well-studied regimens: sequential MTX and bolus FU (Marsh *et al*, 1991) and CI FU modulated by LV (Leichman *et al*, 1993). Oxaliplatin was added to the bolus part.

**Figure 1:**
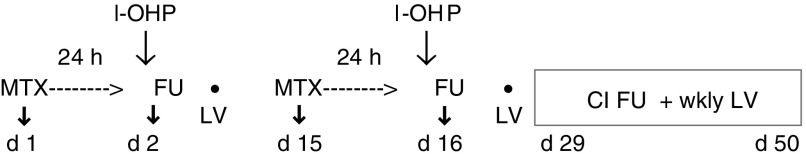
Design of drug regimen. One cycle=8 weeks. In the first part of the regimen, patients were given MTX 200 mg m^−2^ i.v. diluted in 500 ml of D_5_W, infused in 1 h, days 1 and 15; oxaliplatin 85 mg m^−2^ given i.v. as a 2-h infusion in 500 ml of D_5_W followed by FU 600 mg m^−2^ i.v. bolus, days 2 and 16 along with LV rescue (15 mg) given p.o. q 6 h × 6 doses. In the second part of the cycle, patients were given FU 200 mg m^−2^ day^−1^ CI × 3 weeks (from day 29 to 50), modulated by weekly LV (20 mg m^−2^ i.v. bolus). After 1 week of rest, the second cycle was started on day 57.

One complete cycle of treatment consisted of two MTX → l-OHP−FU bolus treatments (200 mg m^−2^ → 85 mg m^−2^ given i.v. as a 2-h infusion in 500 ml of 5% D-glucose (D_5_W) followed by 600 mg m^−2^, respectively) given on days 1, 2 and 15, 16 along with LV rescue (15 mg) given p.o. q 6 h × 6 doses, followed by 3 weeks of CI FU (200 mg m^−2^) given from day 29 to 50, modulated by weekly LV (20 mg m^−2^). After 1 week of rest, the second cycle was started on day 57, provided that the patient had recovered from toxicity. The entire duration of the cycle is thus 8 weeks (Figure 1

). Through implanted catheters and a venous Port-a-cath (Pharmacia) connected to a portable programmable external pump (CADD-1, Pharmacia) or disposable elastomeres (Baxter) CI FU was administered. The infusional cassettes or elastomeres were changed weekly if no toxicity developed earlier.

Toxicity was evaluated on days 1, 15, 29, 36, 43, 50 and 57. Complete blood counts were obtained on the same days. Liver function tests, blood urea nitrogen, creatinine and electrolytes were obtained monthly.

Dose modification criteria for the MTX → l-OHP−FU regimen were as follows: no dose reduction for gastrointestinal grade I and II toxicity. For grade III diarrhoea or mucositis, the treatment was delayed until recovery and the doses of MTX, l-OHP and FU of the next cycle were decreased by 50%. The dose was reduced, after recovery, by 50% also for WBC <3000 mm^3^ or platelets <75 000 mm^3^ on the day of recycle. Treatment was discontinued in case of grade IV toxicity.

Upon the first signs of mucositis and/or palmar-plantar dysaesthesia/burning, CI FU was discontinued and resumed when these symptoms abated. In the case of severe (grade III) mucositis, the infusion was resumed at a reduced FU dose (50%). The dose of LV during the infusional treatment was not modified in this study. Toxicity is expressed according to WHO criteria (Miller *et al*, 1981).

### Response evaluation

Patients who received at least 2 months of therapy (one cycle), with adequate pretreatment and follow-up radiographic studies, were considered assessable for response, as were patients who experienced rapid disease progression after at least two courses of bolus FU.

Measurable tumour was defined as a tumour mass that could be clearly measured in two dimensions by adequate imaging techniques and response evaluation was according to the WHO criteria (Miller *et al*, 1981). Indicator lesions were measured at each successive cycle. The baseline tumour areas and their variations at each successive evaluation were expressed in cm^2^.

Progression-free survival (PFS) and overall survival (OS) were measured from the date of registration to the date of disease progression as defined above or to the date of death, respectively and calculated using the Kaplan–Meier method (Kaplan and Meier, 1958). Early progressions and deaths, toxic deaths if any, early withdrawals and deaths from other causes were included as failures.

### Statistical methods

The general philosophy behind this phase II study was that l-OHP should add some further activity to our alternating bolus-infusional modulated FU regimen. The new combination would be regarded as promising if an additional 15% response rate is added to our basic regimen. Since our standard alternating regimen without l-OHP affords 30–35% response rate, we were searching for a range of activity around 40–60% to consider the new combination for a new phase III study.

According to the two-stage Simon's design (Simon, 1989), setting Po=40 and P1=60%, with an alpha error=0.1 (reflecting the chances to accept an ‘inactive’ regimen) and a beta error=0.1 (reflecting the chances of accepting as ‘active’ a truly inactive regimen), the treatment will be discontinued if less than seven responses are observed among the first 18 patients. Otherwise, we will proceed to the second stage, where 22 responses over 46 patients will be necessary to define the study as successful and proceed further with the clinical development of this combination.

## RESULTS

### Patient characteristics

Between December 1998 and January 2001, 46 patients meeting the eligibility criteria were registered from six participating Institutions. Table 1

**Table 1:** Baseline patient characteristics

*N*	46
Median age (range)	63 (40–78)
Male, *n* (%)	31 (67)
Female, *n* (%)	15 (33)
	
ECOG PS, *n* (%)	
0	30 (65)
1	16 (35)
	
Primary tumour site, *n* (%)	
Colon	38 (83)
Rectum	8 (17)
	
Number of organs involved, *n* (%)	
1	26 (57)
2	18 (39)
⩾3	2 (4)
	
Site of metastases, *n* (%)	
Liver	27 (59)
Lung	5 (11)
Peritoneum/nodes/others	14 (30)
	
Prior adjuvant chemotherapy, *n* (%)	13 (28)
	
Median number of lesions measured per patient (range)	3 (1–7)
	
Median baseline tumour area (cm^2^) (range)	21 (1–343)

shows patient characteristics. In all, 91% of patients had had surgery on the primary neoplasm. A total of 13% of patients had received prior adjuvant chemotherapy consisting in six cycles of FU+LV. Lesions were measured by CT scan in 34 patients, the remaining being measured by NMR. The median measured baseline tumour area was 21 cm^2^ (range 1–343).

### Treatment outcome

Five patients were not evaluable for response. This was due to rapid clinical deterioration in one patient not allowing completion of the first cycle of treatment; four patients refused to continue treatment after the first drug administration due to toxicity. According to the intention-to-treat principle, all these patients were included in the analysis of response as failures.

Three complete (CR) and 16 partial (PR) responses were obtained (response rate, 42% on the intention-to-treat basis; 95% confidence limits=28–55); in addition, 30% of patients had stable disease (Table 2

**Table 2:** Response to treatment: intention-to-treat analysis (*n*=46)

Complete responses	3 (7 %)
Partial responses	16 (35%)
Stable disease	14 (30%)
Failures	13 (28%)
	
Response rate (95% CL)	42% (28–55)

). A total of 13 failures were reported: eight patients progressed after the first cycle of treatment and four patients refused to continue treatment. In all, 90% of the cases of progression were due to the appearance of new lesions rather than enlargement of the indicator lesions.

The median time to achieve a PR or CR was 91 days (range 57–186), with initial responses attained after one cycle (12 cases), two cycles (four cases) and three cycles (three cases). Two out of three CR were observed after two cycles.

None of the patients in this study underwent surgical exploration in order to resect residual disease.

Only five of the 19 responses were obtained in patients with multiple metastatic sites, the rest being liver only (11 patients), lung only (one patient) and extra hepatic intra-abdominal disease (two patients).

The combined CR and PR rate was 46 and 31% in patients with an ECOG PS of 0 and 1, respectively.

Age and sex did not appear to influence the overall clinical response.

The median duration of response was 5 months (range 2–18) and the median duration of stable disease was 3 months (range 2–7). All patients are now off treatment.

**Figure 2:**
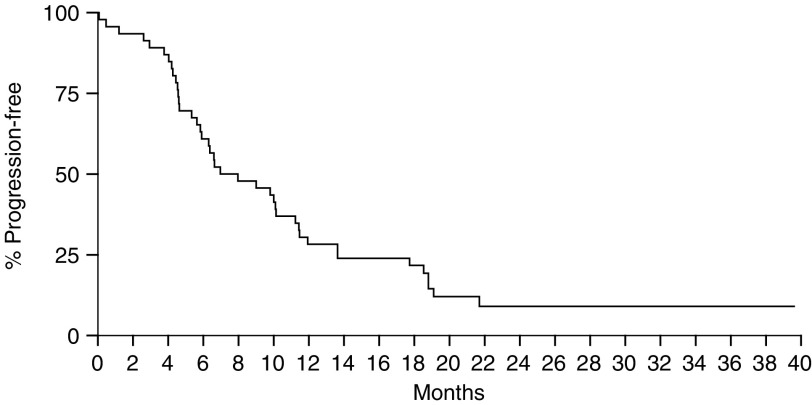
Kaplan–Meier PFS curve for all 46 patients.

**Figure 3:**
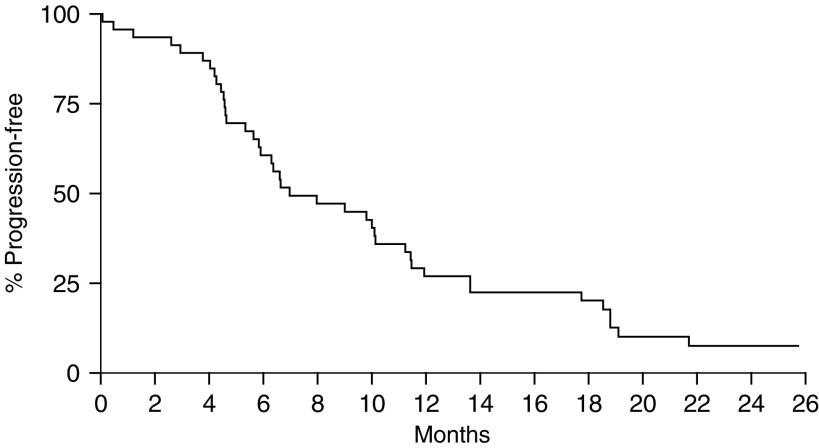
Kaplan–Meier survival curve for all 46 patients.

Two patients declined further chemotherapy while they were still responding; they were considered treatment failures as of the date the treatment was discontinued. After a median follow-up time of 21 months, all patients have progressed and 16 patients are still alive. The median PFS was 6.9 months (Figure 2

) with an OS of 15.9 months (Figure 3

).

### Safety

A total of 112 cycles of treatment (2 months each) were administered, with a median of two cycles (range 0–4) per patient.

Table 3

**Table 3:** Toxicity: worst WHO grade per patient across all cycles (*n*=46)

	**MTX → l-OHP−FU toxicity grade (%)**				**CI FU+6-S-LV toxicity grade (%)**			
**Toxicity**	**I**	**II**	**III**	**IV**	**I**	**II**	**III**	**IV**
Mucositis	15	9	9	2	19	11	2	0
Diarrhoaea	13	4	7	0	11	6	2	0
Nausea/vomiting	32	11	9	0	28	9	0	0
Neurotoxicity	43	10	0	0	0	0	0	0
Conjunctivitis	15	0	0	0	17	2	0	0
Hand–foot syndrome	4	2	0	0	30	4	0	0
Haematological	28	9	0	4	28	19	6	0

reports the worst toxicity of each type, suffered by each patient, across all cycles. The two parts of the regimen are considered separately. No toxic deaths were reported following either part of the regimen.

Mucositis was the most common severe side effect: 11% of patients in the bolus part and 2% in the CI part experienced grade III–IV. Nausea and vomiting were the most frequent side effects in both parts: 9% of patients experienced grade III nausea and vomiting, while no grade III was reported in the CI part.

A total of 53% of patients experienced grade I or II neurotoxicity after l-OHP administration. In most cases, this side effect was limited to the 2–3 days after chemotherapy and recovered between cycles. No grade III or IV was reported.

Hand–foot syndrome was almost exclusive of the CI part with an incidence of 30% of grade I and 4% of grade II.

No major catheter-related complications have been reported in this series of patients coming from centres with long-standing experience in protracted infusion of FU.

## DISCUSSION

Our study shows that high activity can be obtained at the cost of low toxicity using this original but complicated schedule of four drugs.

This study is original under two aspects: first, it is the logic extension of our series of studies on schedule-selective biochemical modulation as mentioned in the introduction (Sobrero *et al*, 1993, 1995, 2000, 2001; Aschele *et al*, 1998); second, it shows the feasibility of combining sequential MTX → FU with l-OHP. The question we are focusing on now is where to place this regimen in the scenario of advanced colorectal cancer treatment with fluoropyrimidines–l-OHP combinations. Table 4

**Table 4:** Activity and safety (grade III–IV toxicity) of fluoropyrimidines–l-OHP combinations as first-line treatment of advanced colorectal cancer

**Author**	**Year**	**Regimen**	**Phase**	**No. of patients**	**l-OHP (mg m^−2^)**	**DI (mg m^−2^ week^−1^)**	**RR (%)**	**Nausea/vomiting**	**Mucositis**	**Diarrhoea**	**Neutro**	**Neuro**
de Gramont	2000	FOLFOX4	III	210	85 q 2	42.5	49.5	11.5	5.6	11.9	41.7	18.2
Goldberg	2003	FOLFOX4	III	269	86 q 2	42.5	40	9	NA	14	52	18
Andre'	2003	FOLFOX4	III	312	87 q 2	42.5	58	4	2	9	26	13
Andre'	2003	FOLFOX7	III	313	130 q 2	65	64	7	4	9	20	13
Grothey	2002	FUFOX	III	118	50 q 1	40	48.3	6.8	2.5	21.2	6,7	12.7
Ravaioli	2002	Bolus FU LV 1->5+l-OHP	II	45	130 q 3	43	43.9	15.6	2.2	28.9	20	2.2
Hochster	2003	Wkly bolus FU LV+l-OHP	II	42	85 q 2	42.5	63	2	2	29	10	12
Van Cutsem	2003	XeloOx (Cape+l-OHP)	II	96	130 q 3	43	55	13	NA	16	7	16
Grothey	2003	CapOx (Cape+l-OHP)	II	82	70 d 1.8 q 3	46.6	49.3	2.6	NA	14.3	5.2	5.2
Bennouna	2003	Tegafox (UFT–LV+l-OHP)	II	64	130 q 3	43	32	9	NA	15	8	0
Present paper		MTX–FU–LV+l-OHP	II	46	85 q 2	21.25	42	9	11	7	4	0

DI=dose intensity; RR=response rate; NA=not available.

reports the main features of these regimens in phase II and III studies on advanced colorectal cancer. Four fluoropyrimidine schedules have been combined with the DACH compound: daily bolus × 5 (Ravaioli *et al*, 2002), weekly bolus (Hochster *et al*, 2003), infusional FU (de Gramont *et al*, 2000; Grothey *et al*, 2002; André *et al*, 2003; Goldberg *et al*, 2003) and the oral route using capecitabine or UFT (Bennouna *et al*, 2003; Grothey *et al*, 2003; Van Cutsem *et al*, 2003). Oxaliplatin has either been used at 50 q 1, 85 q 2 or 130 q 3 or 4 weeks with a calculated planned dose intensity of approximately 40–45 mg m^−2^ week^−1^ in all these studies except FOLFOX7, which is intended to deliver 50% higher dose intensity. Very satisfactory response rates have been reported in all these trials (range 32–64%) and considering that the number of patients accrued in each trial is high (range 42–313, median 118), the 95% CI on these values is narrow. Under these conditions, toxicity and convenience play a major role in the selection of the ‘best’ regimen.

While the incidence of mucositis was very low in all the reports, diarrhoea was almost prohibitive with the bolus FU regimens and very relevant with the oral fluoropyrimidine combinations, whereas neutropenia was very common (40–50%) with the classical FOLFOX4. The incidence of neurotoxicity is hard to comment because it was dependent on the cumulative l-OHP dose and this figure is not available in the great majority of the reports. Our study employs half as much l-OHP per cycle: in fact the dose intensity would be 42.5 mg m^−2^ week^−1^ during the month of treatment with MTX → FU+l-OHP, but this figure is halved by alternating with 1 month of infusional FU without l-OHP. It is thus not surprising that our regimen is the only one without high-grade neurotoxicity, making it a potential candidate for prolonged chemotherapy programme. The whole spectrum of toxicity is also particularly favourable with low values all across. One would thus be tempted to further pursue this strategy into phase III. In designing the study we set the Po value at 40% response rate. The results are therefore just in the range of uncertainty whether to bring this regimen to a phase III study or to drop it. On the one hand, the very low toxicity is encouraging for further testing and on the other hand, equiactive but more simple combination regimens with oral fluoropyrimidines or infusional FU plus l-OHP or irinotecan are available. This is particularly true considering the relatively good prognostic factors of our patient population. Consequently, our overall interpretation of these data is that they are not good enough for further studies in the light of the complexity of the regimen. Much more substantial improvement in activity would be needed (P1 was in fact set at 60%, expecting a PFS of approximately 10 months) before embarking onto a randomised phase III trial. For this reason, we are now accruing patients on a similar regimen incorporating both CPT-11 and l-OHP.
